# Neuronal and Muscle Differentiation of Mammalian Cells Is Accompanied by a Change in PHF10 Isoform Expression

**DOI:** 10.1134/S1607672923700643

**Published:** 2024-01-08

**Authors:** D. O. Bayramova, A. M. Azieva, A. V. Feoktistov, S. G. Georgieva, N. V. Soshnikova

**Affiliations:** 1 Department of Transcription Factors, Engelhardt Institute of Molecular Biology, Russian Academy of Sciences, Moscow, Russia; 2 Center for Precision Genome Editing and Genetic Technologies for Biomedicine, Engelhardt Institute of Molecular Biology, Russian Academy of Sciences, Moscow, Russia; 3 National Research Center “Kurchatov Institute”, Moscow, Russia

**Keywords:** neural differentiation, chromatin remodeling, SWI/SNF, PBAF, PHF10, gene expression

## Abstract

The PBAF chromatin remodeling complex of the SWI/SNF family plays a critical role in the regulation of gene expression during tissue differentiation and organism development. The subunits of the PBAF complex have domains responsible for binding to N-terminal histone sequences. It determines the specificity of binding of the complex to chromatin. PHF10, a specific subunit of the PBAF complex, contains a DPF domain, which is a unique chromatin interaction domain. A PHF10 isoform that lacks the DPF domain is also present in vertebrate cells. This work shows that during neuronal and muscle differentiation of human and mouse cells, the expression of PHF10 isoforms changes: the form that does not have DPF replaces the form in which it is present. Replacement of PHF10 isoforms in the PBAF complex may affect its selectivity in the regulation of genes in differentiating cells.

Chromatin remodeling is performed by special complexes that change the position of nucleosomes relative to DNA using the energy of ATP hydrolysis. Among the latter, the SWI/SNF family of complexes is involved in the regulation of gene expression [1]. Knockout of the subunits of the complex is lethal for the organism or leads to defects in the development of certain organs [2]. The SWI/SNF family includes three types of complexes (BAF, PBAF, and GBAF), which differ in subunit composition [3]. The complexes contain 10–12 subunits, some of which are common, while others are specific to each of the complexes. A number of subunits of the complexes have domains that recognize various modifications of the N-terminal histone sequences, which determines the interaction of the complex with a certain chromatin region [4].

The PBAF complex differs from other complexes of the family by the presence of a chromatin-binding module consisting of four proteins: BAF200, BAF180, BRD7 and PHF10. Each of these proteins contains domains that recognize histone N-terminal modifications, allowing the complex to be specifically localized on chromatin.

The PHF10 subunit plays an important role in the functioning of the complex, since knockout of the PHF10 gene in mice is embryonic lethal [5]. PHF10 has an internal structured part consisting of a WHD domain and two alpha helices (Figs. 1a, 1b).

**Fig. 1.**
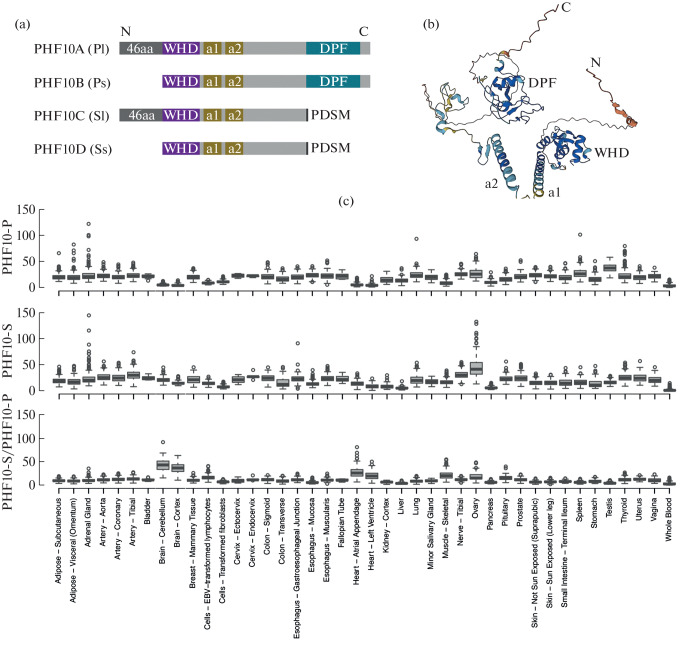
(a) Scheme of PHF10 isoforms. The domains and motifs of PHF10 isoforms are indicated. (b) Predicted structure of PHF10-Pl (Q8WUB8) according to Alpha Fold database (https://alphafold.ebi.ac.uk/entry/Q8WUB8). (c) Expression of PHF10-P and PHF10-S isoforms and their ratio in various human tissues according to the GTEx database (the genotype-tissue expression (GTEx) project data). The *Y* axis shows TPM (transcripts per million reads in the sample).

We previously showed that PHF10 has isoforms that differ in the N- and C-termini (Fig. 1a) [6]. As a result of start from alternative promoters, protein isoforms may contain (PHF10-Pl and PHF10-Sl) or not contain (PHF10-Ps, PHF10-Ss) the N-terminal 46 amino acids. As a result of alternative termination, the C-terminus of the isoforms ends with a DPF domain (PHF10-Pl and PHF10-Ps, generic name PHF10-P) or, instead, with a SUMO1 conjugation site, PDSM motif (PHF10-Sl and PHF10-Ss, generic name PHF10-S). DPF belongs to the group of PHD domains that interact with N-terminal histone sequences. DPF is a unique domain found only in several proteins [7]. According to modeling, the DPF domain of the PHF10 protein is able to bind to the H3K14ac N-terminus of histone [8]. H3K14ac modification is characteristic of activated genes and is localized on the promoters and coding part of genes [9].

In addition to different domain organizations, the isoforms have different phosphorylation patterns. The PHF10-Pl and PHF10-Ss isoforms differ from each other most significantly (Fig. 1a).

During myeloid differentiation, PHF10-P are recruited to promoters of specific genes [10] and are involved in the recruitment of RNA polymerase II [6]. In addition, PHF10-P isoforms were shown to play an important role in maintaining the proliferation of neuronal progenitors and fibroblasts [11, 12]. Much less is known about the role of PHF10-S isoforms. We established that they are included in the PBAF complex alternatively to PHF10-P isoforms, are phosphorylated at other amino acid residues, and are therefore much more stable than PHF10-P isoforms [13]. The majority of cell lines of oncogenic origin express both PHF10-P and PHF10-S isoforms, which makes it difficult to study their functions.

In this work, we found that, of all human tissues, nervous and muscle tissues are most enriched in PHF10-S isoforms. We also showed that, when cell lines differentiate along the neuronal and muscle pathways, PHF10-Ss isoforms begin to be expressed, whereas the expression of PHF10-Pl isoforms significantly decreases. This phenomenon of “isoform expression switching” is a conserved process that is observed in human and mouse neuronal cell cultures.

To study the functional characteristics of the isoforms, we analyzed the expression of DPF-containing and not containing isoforms and their ratio in various human tissues presented in the GTEx (The Genotype-Tissue Expression Project data) database (Fig. 1b) [14]. Both types of isoforms are expressed in almost all tissues at approximately the same level. However, in brain tissue (cerebral cortex and cerebellum on the plot), heart (atrium and left ventricle on the plot), and muscle tissue (skeletal muscle), the expression of DPF-free isoforms (PHF10-S) predominates.

Since it was previously shown that the DPF-containing form is required for cell proliferation, we assumed that its replacement in tissues with a DPF-free isoform may be associated with cell differentiation. To further study the relationship between these processes, several cell lines were selected and differentiated. The human SH-SY5Y line of neuronal origin was differentiated for 3 days using ATRA (all-*trans* retinoic acid) (10 μM) followed by the addition of BDNF factor (50 ng/mL) in neurobasal medium supplemented with 1× B27 and Glutamax. Throughout differentiation, changes in cell phenotype and gradual formation of neurites were recorded using light microscopy (Fig. 2b, left panel). We also selected cells, lysed them, and performed Western blotting analysis using staining with antibodies to PHF10, which recognize all PHF10 isoforms that we had obtained previously [6]. Protein amounts were aligned to tubulin expression (Fig. 2a, right panel). As early as on day 5, we detected a decrease in the expression of the DPF-containing isoform PHF10-Ps and an increase in DPF-free isoform PHF10-Sl. In addition, the PHF10-Ss isoform, which was absent in undifferentiated cells, began to be expressed. On day 10, the expression of PHF10-Pl was not detected, the expression of PHF10-Ps became even less, whereas the expression of PHF10-Ss significantly increased (Fig. 2a, right panel). Thus, during neuronal differentiation of human cells, we observed a change in the expression of PHF10 isoforms: PHF10-Pl ceased to be expressed, whereas PHF10-Ss began to be expressed and, together with PHF10-Sl, became predominant in differentiated SH-SY5Y cells.

**Fig. 2.**
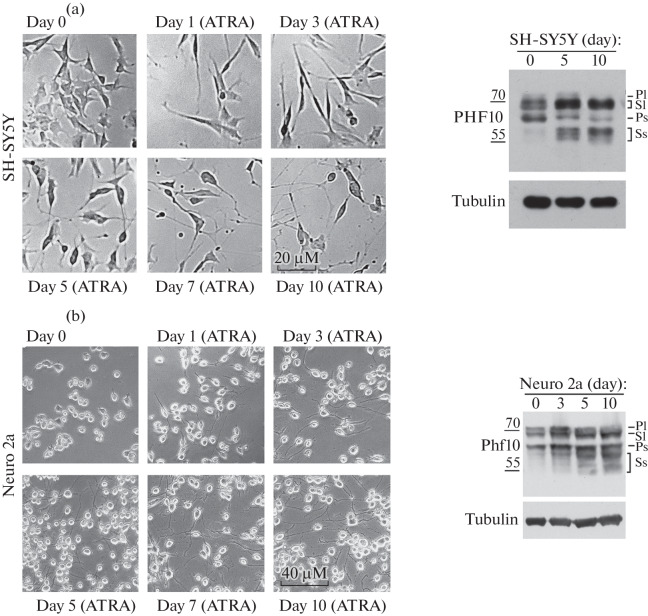
(a, b) Neuronal differentiation of human SH-SY5Y cells (a) and mouse Neuro 2a cells (b): left panel—visualization using negative contrast of cell phenotype during 10 days of differentiation; right panel—Western blot analysis of PHF10 isoforms without differentiation, in the middle and at the end of differentiation. Isoforms are indicated on the right. Molecular weight markers (55 and 70 kDa) are shown on the left. Staining with antibodies against tubulin was used as a loading control.

Differentiation of another line of neuronal origin, mouse Neuro2A cells, was performed for 10 days using ATRA. The phenotype of these cells was somewhat different from that of SH-SY5Y; however, they also produced neurites and stopped dividing by the endo of differentiation (Fig. 2b, left panel). Expression of Phf10 isoforms was also detected by Western blotting and staining with anti-Phf10 antibodies (Fig. 2b, right panel). On day 10 of differentiation, we also observed a cessation of expression of the Phf10-Pl isoform, an increase in the expression of the Phf10-Sl isoform, and a pronounced expression of the Phf10-Ss isoform. However, unlike SH-SY5Y, the expression of Phf10-Ps did not change, which may be due to the fact that some processes and signaling pathways in Neuro2A cell culture may differ from in vivo differentiation. In general, the trend of changing the expression of Phf10-Pl isoforms to Phf10-Ss was also observed in these mouse cells, which indicates the conservation of the molecular mechanisms regulating the “switching of isoforms” and the importance of PHF10-Ss isoforms in the nervous tissue of mammals.

The immortalized mouse skeletal myoblast line C2C12 was also differentiated along the muscle pathway by replacing 20% bovine serum with 1% horse serum in the cell culture medium [15]. Using Western blotting, changes in the expression levels of Phf10 isoforms were detected as early as on days 3 and 5 of differentiation. The balance of isoforms shifted towards the isoforms that do not contain DPF: the expression of Phf10-Pl ceased, whereas the level of Phf10-Ss and Phf10-Sl isoforms increased (Fig. 3).

**Fig. 3.**
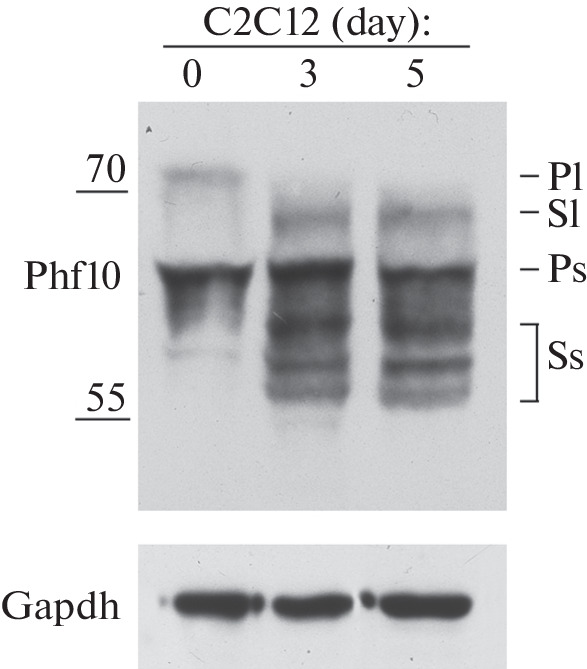
Western blot analysis of Phf10 isoforms during myogenic differentiation of C2C12 mouse cells. Isoforms are indicated on the right. Molecular weight markers (55 and 70 kDa) are shown on the left. Staining with antibodies against the Gapgh protein was used as a loading control.

Thus, during neuronal and myogenic differentiation of mammalian cells (mouse and human), the expression of PHF10 isoforms changes. The isoforms that do not contain DPF replace the isoforms with the DPF domain. In particular, PHF10-Pl ceases to be expressed and is replaced by the PHF10-Ss isoform, which is not detected in undifferentiated cells but becomes predominant in terminally differentiated cells.

Cell differentiation is accompanied by changes in transcribed genes: their epigenetic modifications, chromatin structure, and spatial organization change. This leads to the fact that the expression of some genes (in particular, proliferation genes), is inhibited, whereas the expression of tissue-specific genes is activated. It was previously been shown that some PBAF subunits have homologues that are expressed in certain cells and tissues [3]. Thus, the composition of SWI/SNF complexes may be specific to certain cell types.

PHF10 isoforms are alternatively included in the PBAF complex and impart specificity to the complex with respect to the chromatin with which the complex preferentially binds. The DPF domain of PHF10-P isoforms potentially binds to the H3K14ac histone N-terminus [8]. H3K14ac is localized on the promoters and coding part of genes and appears on tissue-specific genes during their de novo activation [9]. The PHF10-Ss isoform does not contain this domain; however, instead of this domain, it is able to covalently conjugate to SUMO1 [6]. SUMO1 can bind to SIM motifs (Sumo-interacting motifs) of other proteins. In addition, PHF10-Pl and PHF10-Ss have different phosphorylation patterns: in PHF10-Pl, more than ten serine and threonine residues are phosphorylated in the unstructured part of the N-terminus of PHF10, whereas in PHF10-Ss the unstructured part closer to the C-terminus undergoes such intense phosphorylation [13]. Various modifications may also contribute to the selectivity of the PBAF complex, including different isoforms, with respect to interacting proteins and transcription factors.
